# Association of food insecurity with changes in diet quality, weight, and glycemia over two years in adults with prediabetes and type 2 diabetes on medicaid

**DOI:** 10.1038/s41387-024-00273-7

**Published:** 2024-04-09

**Authors:** Kristine D. Gu, Jessica Cheng, Vicki Fung, Douglas E. Levy, Sydney McGovern, Jessica L. McCurley, Cheryl R. Clark, Anne N. Thorndike

**Affiliations:** 1 Division of Endocrinology, Massachusetts General Hospital, Boston, MA USA; 2 Harvard Medical School, Boston, MA USA; 3 Division of General Internal Medicine, Massachusetts General Hospital, Boston, MA USA; 4 Department of Epidemiology, Harvard T.H. Chan School of Public Health, Boston, MA USA; 5 Mongan Institute Health Policy Research Center, Massachusetts General Hospital, Boston, MA USA; 6 Department of Psychology, San Diego State University, San Diego, CA USA; 7 Division of General Internal Medicine and Primary Care, Brigham and Women’s Hospital, Boston, MA USA

**Keywords:** Type 2 diabetes, Pre-diabetes

## Abstract

Little is known about longitudinal associations between food insecurity (FI) and diet, weight, and glycemia in people with prediabetes and type 2 diabetes (T2D). In a secondary analysis of Medicaid-enrolled health center patients with prediabetes or T2D in Boston, Massachusetts (*N* = 188), we examined associations between food security (FS) and measures of diet quality, weight, and hyperglycemia. FS (10-item USDA FS module) was ascertained at baseline, 1-year, and 2-year follow-up and categorized as persistently secure, intermittently insecure, or persistently insecure. Associations between FS category and changes in Healthy Eating Index-2020 (HEI-20), body mass index (BMI), and hemoglobin A1c (A1c) from baseline to year 2 were assessed using multivariate generalized linear models. Participants had median (p25, p75) age of 52 (42, 57); 71.8% were female and 62.8% Hispanic. Over follow-up, 32.4% were persistently food secure, 33.0% intermittently insecure, and 34.5% persistently insecure. Baseline mean (SD) HEI-20, BMI, and A1c were 55.8 (14.5), 35.9 (8.7) kg/m^2^, 7.1% (1.6) and did not differ by FS category. FS category was not associated with changes in HEI-20, BMI, and A1c at 2 years (all *p* > 0.05). Results suggest that Medicaid-enrolled adults with prediabetes or T2D, regardless of FS status, would benefit from dietary and weight management interventions.

## Introduction

Food insecurity (FI) disproportionately affects people with prediabetes and diabetes [1] and is associated with poor glycemic control [2]. While mechanisms explaining the relationship between FI and hyperglycemia remain unclear, diet may play a role. Poor diet quality has been associated with higher hemoglobin A1c (A1c) among adults with diabetes both cross-sectionally [3] and longitudinally [4].

Most research assessing FI and diabetes has included people with variable access to health care, including individuals with and without health insurance, and controlling for access to adequate medical care is challenging. In the current study, we sought to examine the association of 3 food security (FS) categories over 2 years (persistently secure, intermittently insecure, and persistently insecure) and 2-year changes in diet quality, weight, and glycemia in adults with prediabetes and type 2 diabetes (T2D) who had stable access to health care (i.e., enrolled in Medicaid with established care in a community health clinic). We hypothesized that those who were persistently food insecure over 2 years would have greater decrease in diet quality and higher increase in body mass index (BMI) and A1c over 2 years compared to those who were intermittently insecure or persistently secure.

## Methods

This was a secondary analysis of data from LiveWell, a cohort study to evaluate the impact of Massachusetts Flexible Services (Flex), a Medicaid-funded program for accountable care organizations (ACO) to partner with community-based social service organizations to provide nutrition and housing support for ACO enrollees. Flex eligibility included positive screening for food or housing insecurity and presence of a complex physical or behavioral health need (e.g., obesity, uncontrolled diabetes, uncontrolled depression), high emergency department use (i.e., ≥2 visits in 6 months or ≥4 visits in 1 year), or high-risk pregnancy [5]. LiveWell participants were recruited between December 2019 and December 2020 from 5 community health centers affiliated with a large health system in Boston, Massachusetts. Eligible patients were 21-62 years old, had ≥2 health center visits in the prior 2 years, and spoke English or Spanish; 846 patients with Medicaid insurance were enrolled. LiveWell study procedures were approved by the Mass General Brigham institutional review board on August 27, 2019 and all participants provided verbal informed consent for participation.

This analysis included 188 LiveWell participants with prediabetes or T2D at enrollment. Diagnoses were determined using the electronic health record (EHR) to identify an International Classification of Diseases-10 code, problem list diagnosis, use of T2D medication(s), or a laboratory value consistent with prediabetes or diabetes within 2 years prior to enrollment. When the diagnosis was unclear (i.e., EHR diagnosis of prediabetes or T2D but no prior abnormal A1c), a physician (K.D.G.) performed chart review to adjudicate the diagnosis.

Baseline and annual follow-up surveys collected data on gender, race/ethnicity (baseline only), marital status, number of dependents, household income, health insurance, height and weight, Patient Health Questionnaire-8 [6], Generalized Anxiety Disorder-7 [7], housing instability, financial stress, cost-related medication underuse [8], and FS status. FS was assessed with the USDA 10-item Adult FS Survey Module and was dichotomized (≤ 2 = FS; 3-10 = FI) [9]. Participants who indicated FS at all 3 timepoints (baseline, 1-year, and 2-year follow-up) were coded as “persistently secure,” 1 or 2 timepoints as “intermittently insecure,” and no timepoints as “persistently insecure.

Primary outcomes were changes in Healthy Eating Index-2020 (HEI-20) scores, BMI, and A1c over 2 years, calculated as the value at year 2 minus the value at baseline for each outcome. HEI-20, a valid and reliable measure of dietary quality [10] that aligns with the Dietary Guidelines for Americans 2015-2020 [11], was calculated from 2 Automated Self-Administered 24-Hour dietary recalls collected at baseline and annual follow-up using the National Cancer Institute’s simple scoring algorithm [12]. Scores range from 0 (least healthy) to 100 (most healthy). BMI (kg/m^2^) was calculated using self-reported weight and height from annual surveys because EHR data was less complete due to pandemic-related virtual visits. Hemoglobin A1c was ascertained using EHR-recorded laboratory data. We used the average A1c within 24 months preceding enrollment to represent baseline A1c, and average A1c within 12 months preceding the 2-year follow-up survey to represent year 2 A1c. We chose this strategy to capture average exposure to hyperglycemia leading up to enrollment and 2-year follow-up dates.

Baseline differences in participant characteristics by FS category (persistently secure, intermittently insecure, persistently insecure) were compared using non-parametric tests. Separate multivariate generalized linear models (Gaussian distribution with identity link) adjusted for age, gender, and ethnicity were used to test the association of FS category with change in each outcome. The assumptions of generalized linear models were met. Those missing FS data at any of the 3 time points were excluded (*N* = 37). Participants who were missing HEI-20 (*N* = 3), BMI (*N* = 73), and A1c (*N* = 56) at baseline or year 2 were also excluded from the relevant models. To address missing data on BMI, a sensitivity analysis was conducted using available EHR weight data to impute missing self-reported weights. All analyses were run using SAS 9.4 (Cary, NC).

## Results

Among 188 adults with prediabetes and T2D, 61 (32.4%) were persistently food secure, 62 (33.0%) intermittently insecure, and 65 (34.5%) persistently insecure. Participants had median (p25, p75) age of 52 (42, 57), 71.8% were female, and 62.8% Hispanic. There were baseline differences between groups in ethnicity, housing instability, financial stress, cost-related medication underuse, and depression and anxiety symptoms (Table 1). The baseline unadjusted mean (SD) HEI-20 score, BMI, and A1c were 55.8 (14.5), 35.9 (8.7) kg/m^2^, 7.1% (1.6), respectively.

**Table 1:** Baseline characteristics of participants with prediabetes and type 2 diabetes by food security category.

	All participants (*N* = 188)	Persistently secure (*N* = 61)	Intermittently insecure (*N* = 62)	Persistently insecure (*N* = 65)	*p*-value
**Sociodemographic characteristics**					
Age, median (p25, p75)	52.0 (42, 57)	51.0 (41, 58)	53.5 (42, 57)	51.0 (43, 57)	0.92
Female sex, N (%)	135 (71.8)	42 (68.9)	42 (67.7)	51 (78.5)	0.33
Race, N (%)					0.5
Black	30 (16.4)	9 (15.0)	13 (21.7)	8 (12.7)	
More than 1 race^a^	26 (14.2)	8 (13.3)	7 (11.7)	11 (17.5)	
Other races^b^	50 (27.3)	13 (21.7)	16 (26.7)	21 (33.3)	
White	77 (42.1)	30 (50.0)	24 (40.0)	23 (36.5)	
Ethnicity					
Hispanic	118 (62.8)	34 (55.7)	35 (56.5)	49 (75.4)	0.03
Not Hispanic	70 (37.2)	27 (44.3)	27 (43.6)	16 (24.6)	
High school education or less, N (%)	88 (46.8)	28 (45.9)	32 (51.6)	28 (43.1)	0.62
Married/living with significant other, N (%)	69 (37.5)	22 (36.1)	23 (38.3)	24 (38.1)	0.96
Any dependents, N (%)	90 (48.6)	30 (49.2)	27 (43.5)	33 (53.2)	0.56
Participated in SNAP, N (%)	126 (67.0)	36 (59.0)	46 (74.2)	44 (67.7)	0.2
**Health-related social needs**					
Housing instability, N (%)	64 (34.2)	14 (23.0)	18 (29.5)	32 (49.2)	<0.01
Financial stress, moderate/severe, N (%)	50 (26.6)	2 (3.3)	18 (29.0)	30 (46.2)	<0.01
Cost-related medication underuse, N (%)	28 (15.0)	2 (3.3)	14 (23.0)	12 (18.5)	<0.01
**Clinical and behavioral characteristics**					
Current smoking, N (%)	35 (18.6)	11 (18.0)	7 (11.3)	17 (26.2)	0.01
Hypertension, N (%)	103 (54.8)	29 (47.5)	37 (59.7)	37 (56.9)	0.37
Diabetes status					0.39
Prediabetes	76 (40.4)	29 (47.5)	23 (37.1)	24 (36.9)	
Type 2 diabetes	112 (59.6)	32 (52.5)	39 (62.9)	41 (63.1)	
Anti-hyperglycemic medications, N (%)					0.07
None	81 (43.1)	33 (54.1)	26 (41.9)	22 (33.9)	
Non-insulin medications	61 (32.5)	14 (23.0)	18 (29.0)	29 (44.6)	
Insulin	46 (24.5)	14 (23.0)	18 (29.0)	14 (21.5)	
Physical activity (total metabolic equivalent of task per day), median (p25, p75)	1392.5 (313.5, 3682.5)	1455.0 (339.0, 3546.0)	996.0 (198.0, 3450.0)	1825.5 (281.5, 3939)	0.56
PHQ-8 score, Median (p25, p75)	7.5 (3, 13)	3.0 (1, 8.5)	7.0 (4, 15)	11.0 (8, 14)	<0.01
GAD-7 score, Median (p25, p75)	6.0 (2, 12)	2.0 (0, 6)	6.5 (3, 14)	9.0 (5, 14)	<0.01
No. A1c measurements at baseline, median (p25, p75)	2 (1, 4)	2 (1, 3)	3 (1, 4)	2 (1, 4)	0.3
No. A1c measurements at year 2, median (p25, p75)	2 (1, 2)	1 (1, 2)	2 (1, 2)	2 (1, 3)	0.21

*SNAP* Supplemental Nutrition Assistance Program, *PHQ*-*8* 8-item Patient Health Questionnaire, *GAD*-*7* 7-item Generalized Anxiety Disorder questionnaire, *A1c* hemoglobin A1c

^a^Includes participants who selected more than 1 race (*N* = 6) and self-identified “mixed” race (*N* = 20)

^b^Includes Asian (*N* = 3), Native American or Alaskan Native (*N* = 1), and self-identified “other” race (*N* = 46)

Note: Kruskal Wallis test was used for continuous variables and Chi-square or Fisher’s exact tests for categorical variables.

**Fig. 1:**
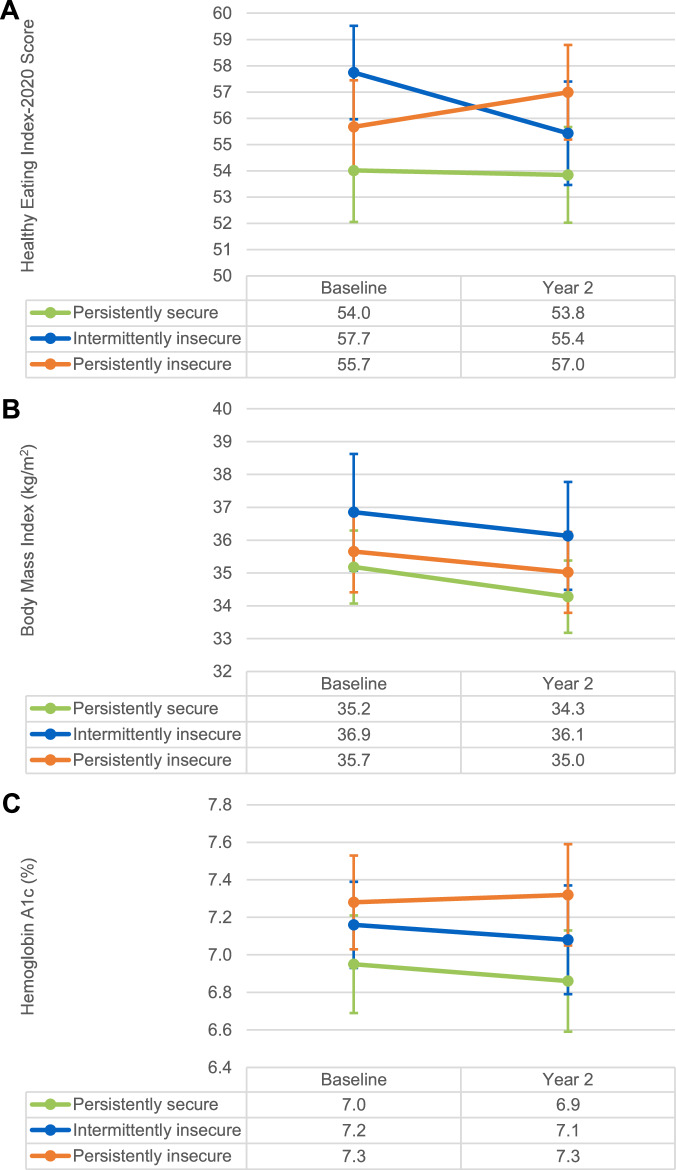
Unadjusted means and standard errors for outcomes at baseline and year 2 by food security category. **A** Healthy Eating Index-2020 score; (**B**) body mass index; (**C**) hemoglobin A1c.

Figure 1 shows unadjusted mean HEI-20, BMI, and A1c by FS category at each timepoint. Unadjusted mean A1c was higher in the persistently insecure versus persistently secure groups at baseline (7.3 vs. 7.0%) and year 2 (7.3 vs. 6.9%), but there was no evidence that the 2-year change in HEI-20, BMI, or A1c differed by FS category adjusting for age, gender, and ethnicity (all p-values > 0.05, Supplementary Table 1). Results were similar when using a mixed model. A sensitivity analysis using imputed BMI yielded similar results (Supplementary Table 1).

## Discussion

In this sample of Medicaid recipients with prediabetes and T2D, nearly two-thirds reported FI at least once during the 2-year study period. Those who had persistent FI were more likely to have other health-related social needs, including housing instability and financial stress, compared to those who were intermittently food insecure or persistently food secure. Contrary to our hypothesis, we did not find differences in change in diet quality, BMI, or A1c by the degree of persistence of FI.

Longitudinal studies examining the association between FI and glycemic control in adults with prediabetes and T2DM are limited. While some studies have demonstrated worse glycemic control in those with FI, there is limited evidence that this disparity widens over time. A study of participants from 4 clinics affiliated with an academic medical center found that FI was associated with higher A1c at baseline (7.6 vs. 7.0%), a difference that remained constant over a mean follow-up of 37 months [13]. Another study found that among adults with T2D receiving care at a federally qualified health center with access to the same comprehensive diabetes management program, those with FI vs. FS had higher A1c at baseline (9.11 vs. 8.56%) and through 2 years of follow-up [14], but they unexpectedly found that those with FI had a significantly greater improvement in A1c over 2 years. In a larger study of almost 3 000 Medicare patients at an integrated health delivery system, those with FI had higher A1c at baseline (7.4 vs. 7.1%), a difference that remained constant at 1-year follow-up in an unadjusted analyses but was no longer significant after adjusting for sociodemographic and clinical characteristics [15].

In contrast to prior studies, we did not find evidence that FI was associated with higher A1c at baseline. A possible explanation is that access to Medicaid coverage and care from a community health center may have mitigated some of the adverse effects of FI on diabetes management through access to medications and clinical visits. This was supported by similarities in diabetes medication use and number of A1c measurements across FS categories in our study. Furthermore, federal relief funding during the COVID-19 pandemic, including expanded Medicaid and Supplemental Nutrition Assistance Program (SNAP) benefits [16], may have also reduced some of the negative effects of FI on health and diet during our study period. While our findings do not show an association between persistent FI and worsening A1c over 2 years, further research is needed to explore whether differences exist in a larger population or over a longer period than assessed by our study and others. Additionally, future research examining the long-term effect of social disadvantage (e.g., cumulative longitudinal exposure to food insecurity, housing instability, financial stress, and cost-related medication underuse) is needed.

A strength of this study is the longitudinal analysis that included multiple assessments of FS status over time to account for the dynamic nature of FS, which may fluctuate depending on many factors including income, SNAP cycle, or time of year [17]. Study limitations include the small sample size, which may have limited power to detect differences in outcomes. The sample was restricted to adults with Medicaid receiving care in a single health system in Massachusetts, which limits generalizability. Participants were recruited during the early months of the COVID-19 pandemic, which may have impacted health-related social needs, health behaviors, and healthcare utilization [18, 19]. Finally, A1c was collected at various frequencies and time points, and BMI calculations relied on self-reported weight.

In sum, among Medicaid-insured adults with prediabetes and T2D followed over 2 years, glycemia was stable, diet quality remained low, and BMI remained high, regardless of FS category. These findings reinforce the urgent need for interventions to improve dietary intake and weight in all Medicaid patients with prediabetes and T2D.

### Supplementary information

Supplementary Table 1

## Data Availability

The datasets generated during and/or analyzed during the current study are available from the corresponding author on reasonable request.
