# Supplementary material for: Association of food insecurity with changes in diet quality, weight, and glycemia over two years in adults with prediabetes and type 2 diabetes on medicaid
Source: Nutr Diabetes. 2024 Apr 9;14:16. doi: 10.1038/s41387-024-00273-7 (PMC11003964; doi:10.1038/s41387-024-00273-7)
Supplement: Supplementary file 1 — Supplementary Table 1 [file 41387_2024_273_MOESM1_ESM.pdf]

Supplementary Information

Supplementary Table 1: Fixed effects for the multivariate generalized linear models assessing change in HEI-20, BMI, and A1c from baseline to year 2 by food security category

	Estimate	Standard Error	p-value
**HEI-20**			
Intercept	2.69	6.18	0.66
Food security category			0.39
Persistently secure	Ref		
Intermittently insecure	2.11	2.85	
Persistently insecure	-1.81	2.85	
Age	-0.02	0.11	0.86
Male (ref: female)	-4.32	2.74	0.46
Ethnicity (ref: non-Hispanic)	-0.37	2.57	0.89
**BMI (kg/m^2)**			
Intercept	1.20	2.10	0.57
Food security category			0.93
Persistently secure	Ref		
Intermittently insecure	-0.21	0.99	
Persistently insecure	-0.38	2.10	
Age	-0.01	0.04	0.79
Male (ref: female)	0.08	0.87	0.93
Ethnicity (ref: non-Hispanic)	0.51	0.91	0.57
**Imputed BMI[a] (kg/m^2)**			
Intercept	1.41	1.72	0.41
Food security category			0.99
Persistently secure	Ref		
Intermittently insecure	0.03	0.79	
Persistently insecure	-0.08	0.82	
Age	-0.02	0.75	0.58
Male (ref: female)	0.21	0.75	0.79
Ethnicity (ref: non-Hispanic)	-0.03	0.71	0.97
**A1c (%)**			
Intercept	0.53	0.79	0.50
Food security category			0.88
Persistently secure	Ref		
Intermittently insecure	-0.38	0.32	
Persistently insecure	-0.15	0.32	
Age	-0.01	0.01	0.52
Male (ref: female)	-0.04	0.31	0.90
Ethnicity (ref: non-Hispanic)	0.07	0.29	0.81

[a]Regression modelling was used to examine the relationship between BMI calculated from EHR vs self-reported weights at each timepoint (baseline R^2= 0.97, year 2 R^2=0.94). Using the parameter estimates from the models, we imputed missing self-report data for those with available EHR data (N=36).
